# Biomechanical Role and Motion Contribution of Ligaments and Bony Constraints in the Elbow Stability: A Preliminary Study

**DOI:** 10.3390/bioengineering6030068

**Published:** 2019-08-07

**Authors:** Elisa Panero, Laura Gastaldi, Mara Terzini, Cristina Bignardi, Arman Sard, Stefano Pastorelli

**Affiliations:** 1 DIMEAS-Department of Mechanical and Aerospace Engineering, Politecnico di Torino, c.so Duca degli Abruzzi 24, 10129 Torino, Italy; 2 PolitoBIOMed Lab, DISMA-Department of Mathematical Sciences, Politecnico di Torino, c.so Duca degli Abruzzi 24, 10129 Torino, Italy; 3 PolitoBIOMed Lab, DIMEAS-Department of Mechanical and Aerospace Engineering, Politecnico di Torino, c.so Duca degli Abruzzi 24, 10129 Torino, Italy; 4 Hand Surgery Division, AOU CTO, via Gianfranco Zuretti 29, 10126 Torino, Italy

**Keywords:** elbow instability, posterior medial collateral ligament, coronoid process, biomechanical analysis, motion capture, cluster markers set, specimens

## Abstract

In flexion–extension motion, the interaction of several ligaments and bones characterizes the elbow joint stability. The aim of this preliminary study was to quantify the relative motion of the ulna with respect to the humerus in two human upper limbs specimens and to investigate the constraints role for maintaining the elbow joint stability in different section conditions. Two clusters of four markers were fixed respectively to the ulna and humerus, and their trajectory was recorded by a motion capture system during functional orthopedic maneuver. Considering the posterior bundle of medial collateral complex (pMUCL) and the coronoid, two section sequences were executed. The orthopedic maneuver of compression, pronation and varus force was repeated at 30°, 60° and 90° flexion for the functional investigation of constraints. Ulna deflection was compared to a baseline elbow flexion condition. With respect to the intact elbow, the coronoid osteotomy influences the elbow stability at 90° (deflection = 11.49 ± 17.39 mm), while small differences occur at 30° and 60°, due to ligaments constraint. The contemporary pMUCL section and coronoid osteotomy causes elbow instability, with large deflection at 30° (deflection = 34.40 ± 9.10 mm), 60° (deflection = 45.41 ± 18.47 mm) and 90° (deflection = 52.16 ± 21.92 mm). Surgeons may consider the pMUCL reconstruction in case of unfixable coronoid fracture.

## 1. Introduction

The main biomechanical role of the elbow can be summed up as a cooperation with the shoulder joint for the positioning of the human hand in space. The distal humerus end, the proximal ulna and radius ends define the articular surfaces of the elbow joint. Compared to the shoulder, the elbow allows a more constrained range of motion. Indeed, it is commonly approximated as a hinge joint with one principal rotational degree of freedom (DOF), corresponding to the flexion–extension motion (0–140°) [1,2]. A second DOF consisting in pronation–supination is commonly referred to the wrist joint, even if the elbow partially cooperates during the motion.

The stability of the elbow is provided by osseous, muscular and ligamentous anatomy [3,4]. The elbow stabilizers can be classified as static or dynamic. The static stabilizers consist of humero-ulnar components, medial ulnar collateral ligament (MUCL) and the lateral collateral ligament (LCL) and they can be defined as primary constraints. Considering the primary static stabilizers, the coronoid process is recognized as a key stabilizer against varus stress and it acts at lower and higher flexion degrees [5]. In the elbow flexion angle range 30–70° the ligaments, in particular the MUCL, contribute to joint stability [6]. The latter is composed of (i) the anterior bundle (aMUCL), which is in tension in the range 30–110° of the elbow flexion angle, (ii) the posterior bundle (pMUCL), which is in tension in the range 50–70° of the elbow flexion angle and (iii) the transverse bundle, which does not contribute to elbow stability [7,8]. Secondary static stabilizers include the radial head and the joint capsule [9]. Elbow muscles are dynamic stabilizers. Among them, the lateral extensor musculature (extensor carpi ulnaris, extensor digitorum communis, extensor carpi radialis brevis, extensor carpi radialis longus, anconeus) resists varus forces, while the medial flexor musculature (the flexor carpi ulnaris, flexor carpi radialis, flexor digitorum superficialis, pronator teres) resists valgus forces [2].

In order to investigate interactions and functions between stabilizers, as to classify their contribution, the use of computational modeling may be a promising method. The development of a proper anatomical and biomechanical model of human joints allows simulating several injuries conditions and testing innovative surgical solutions [7,10,11,12,13] with time and cost reduction [14,15,16]. However, in the model development process, both real samples geometry, to perform an anatomical scaling and to characterize the physiological components, and experimental data tests, to impose motions and applied forces, are indispensable. 

Several experimental analyses on cadaveric specimens were conducted to describe the different functions of elbow stabilizers [4,17]. Despite the recognized crucial role of aMUCL, only recent studies have highlighted the importance of pMUCL against elbow dislocation [18]. Shukla, Gluck and colleagues presented several simulations of elbow dislocation [19,20,21,22,23]. In these experiments, the analysis of cadaveric specimens consisted in applying an external rotation moment and valgus force. Moments (2.5–4.5 Nm) and forces (10–25 N) at elbow flexion angles (30°, 60°, 90°) and with several sections of the primary stabilizers were applied. Results assessed the increasing of joint gapping values at proximal (distance between medial epicondyle and proximal sigmoid notch) and distal (distance between medial trochlea and distal sigmoid notch) aspects of the medial ulno-humeral joint and stressed the role of the pMUCL as an elbow stabilizer [19,20,21,22,23]. Despite the significant previous results, even if several section orders of coronoid, aMUCL and pMUCL were considered, the comparison between the section sequences has not already been evaluated. 

A previous pilot study [24] was conducted for analyzing the roles of anterior (aMUCL) and posterior (pMUCL) bundles in elbow stability, and it described a biomechanical measurement based on a functional method for human motion [25,26,27]. One left specimen elbow was investigated in three different conditions: elbow with intact MCL (baseline condition), anterior bundle section, anterior and posterior bundle section. The analysis of ulna deflection relative to the flexion plane during compression, supination valgus and pronation varus maneuver stressed the progressive increase of elbow joint dislocation and the stability functional roles of ligaments in flexion movement.

The main purpose of the present case study deals with the evaluation of the coronoid and pMUCL roles in the maintenance of joint stability comparing different section conditions. The relation between coronoid and pMUCL is investigated through the quantification of the relative motion of the ulna with respect to the humerus, performed using a motion capture-based methodology with clusters of markers. Two cadaveric upper limbs have been considered during experimental tests and two different section sequences of coronoid and pMUCL constraints have been adopted. The anterior bundle was maintained intact. The orthopedic surgeon applied the maneuver for instability investigation at 30°, 60° and 90° of elbow flexion. A standardized biomechanical analysis overcame the anatomical differences in specimens and comparing results.

## 2. Materials and Methods 

### 2.1. Instruments

The instrumentation utilized during the experiments can be summed up as follows:

- 1 self-contained and pre-calibrated Optitrack Bar V120 Trio for 3D motion capture (120 Hz) and markers registration;
- 1 visible light camera for video recording;
- 2 rigid clusters of 4 markers (Ø 12 mm) applied to the humerus and ulna;
- Generic medical instrumentations for specimen preparation.

The Optitrack bar was positioned to cover the 3D working space. The motion capture bar does not require a user calibration due to the pre-calibrated characteristics and the definition of a 3-axis coordinate system of the sensor. The Optitrack bar reference system was assumed as the global reference frame for data acquisition. The visible light camera was positioned to record the video of the entire experiment. The two rigid clusters were specifically designed to accommodate the ulna and humerus and printed with ABS polymer (Stratasys UPrint SE Plus, Stratasys, Eden Praire, MN, USA). Each cluster was made of a base plate with a saddle support for bone fastening and four columns. Markers were fixed on the top of each column.

### 2.2. Specimen Preparation and Surgical Maneuver

Two upper limb specimens were used for the experiments: one female right (Elbow_1) and one male left (Elbow_2) upper limb, both without any trauma evidence at the elbow joint. Before testing, skin, muscles and subcutaneous tissues were removed, while ligaments and tendons were accurately maintained intact. Anthropometric arm lengths were 226 and 277 mm, respectively, for Elbow_1 and Elbow_2, while forearm lengths were 180 and 249 mm, respectively, for Elbow_1 and Elbow_2.

The two clusters of markers were then fixed respectively to the humerus and ulna in correspondence of middle length, where the bone was sufficiently thick. The humerus cluster was also fixed to the support table, to avoid humerus motion during the maneuver. 

Two different analysis were performed. In the first one, with intact elbow and no load applied, the natural elbow flexion–extension from 0° to 90° was registered, where 0° correspond to the elbow completely extended. Secondly, an orthopedic maneuver was applied to the intact elbow for testing the joint instability. The orthopedic maneuver consists in the application of a compression, pronation and varus force to test the varus posteromedial rotatory instability. This strategy, normally adopted by orthopedics for the analysis of elbow instability in vivo, allows for the assessment of joint instability from a functional point of view. The repeatability of gesture and applied forces relied on orthopedic experience. Different cut sequences of elbow stabilizers were then adopted for the two specimens. Elbow_1 was analyzed as intact, with the posterior bundle section, and then with both the posterior bundle section and coronoid osteotomy. Elbow_2 was analyzed as intact, with coronoid osteotomy, and then with coronoid osteotomy and posterior bundle section. In both upper limbs the anterior bundle remained intact. Table 1 resumes the sequences of surgical sections. The maneuver was repeated two times for each surgical condition at 30°, 60° and 90° of elbow flexion.

### 2.3. Data Analysis

A standardized biomechanical method for data acquisition and data analysis was used [24,28]. The definition of two clusters of markers representing ulna and humerus movements and a specific sequence of coordinate system transformations allowed for the definition of a reference regression plane for flexion–extension motion of the intact elbow, and comparison of the ulna trajectory relative to the humerus in several conditions.

#### 2.3.1. Local Reference System Definition

**Figure 1:**
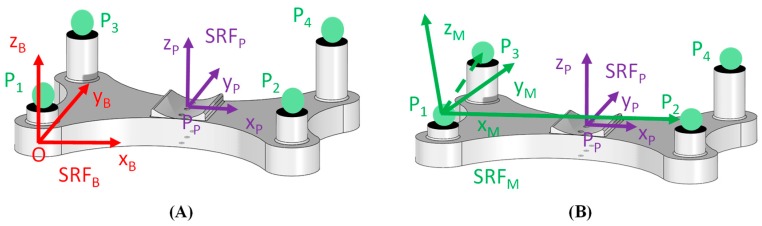
Graphical representation of the spatial reference frames: reference frame of the base plate SRF_B_ and of the bone SRF_P_ (**A**); reference frame of the markers set SRF_M_ (**B**).

Starting from the position of markers on the clusters, a local coordinate system for the ulna and one for the humerus were defined. In Figure 1 a rigid cluster of markers used to track the movement of a bone, either the ulna or humerus, is represented. Firstly, a spatial reference frame of the base plate was defined (SRF_B_) with the origin centered in the middle point of the rigid plate under the smallest support column and the axes were oriented based on plate geometry (Figure 1A). The X_B_ axis was oriented as the longest side of the plate, while Y_B_ axis was oriented as the shortest side of the plate. The Z_B_ axis resulted perpendicular to the previous two axes. The spatial reference frame of the corresponding bone (SRF_P_), either the ulna or humerus, was considered with the origin centered in the middle of the saddle in contact with the bone, and axes oriented as SRF_B_. The saddle shape and the fastening system allowed aligning the axes of the reference frame SRF_P_ to the orientation of the anatomical axes of the bone, with the Y_P_ axis corresponding to the longitudinal bone axis, and X_P_ and Z_P_ axes being the transversal axes. Figure 1A depicts a graphical representation of a marker cluster and the coordinate systems SRF_B_ and SRF_P_.

Secondly, considering three markers, P_1_, P_2_ and P_3_, out of four of the rigid plate and their 3D coordinates with respect to the local SRF_B_, a marker coordinate system SRF_M_ was implemented, as depicted in Figure 1B. The origin was centered in the marker P_1_. X_M_ axis was oriented as the vector P_2_P_1_ and a floating axis was oriented as the vector P_3_P_1_. The cross product of X_M_ axis and floating axis determined the Z_M_ axis and, finally, the Y_M_ axis was obtained by the cross product between Z_M_ axis and X_M_ axis. The fourth marker P_4_ was not considered for reference frame design and it might be useful in case of marker obstruction during motion tracking. Each spatial reference frame can be described by means of a homogeneous matrix and transformation matrixes can be used to represent the relative pose between reference frames [29]. Naming SBRFP the homogeneous matrix of the bone frame with respect to the base frame and SBRFM the homogeneous matrix of the markers frame with respect to the base frame, the transformation matrix TMP which described the rigid relationship that connects markers and the bone coordinate systems was:

TMP=SBRFM−1·SBRFP

During motion tracking recording, the 3D coordinates of the markers P_1_, P_2_ and P_3_ of the base plate, either fixed to the ulna or fixed to the humerus, were measured with respect to the coordinate system of the Optitrack bar. Therefore, the homogeneous matrix of the markers frame with respect to the bar frame, named $S{\mathrm{RF}}_{M}$, was evaluated and, successively, the homogeneous matrix of the bone frame with respect to the bar frame, named $S{\mathrm{RF}}_{P}^{}$, was calculated with the transformation:

SRFP=TMP−1·SRFM

**Figure 2:**
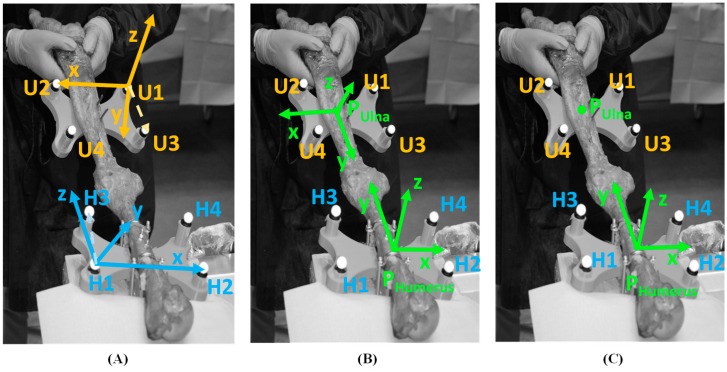
Marker clusters reference systems (**A**); ulna reference frame and humerus reference frame (**B**); ulna origin P_Ulna_ positioning with respect to humerus frame (**C**).

Implementing the above calculus procedure to each rigid cluster of markers, both fixed to the ulna and to the humerus, the homogeneous matrixes of the ulna and of the humerus with respect to the bar frame, named respectively $S{\mathrm{RF}}_{U}^{}$ and $S{\mathrm{RF}}_{H}^{}$, were evaluated. Figure 2A shows markers reference frames for each rigid plate and Figure 2B depicts the bones coordinate systems for ulna and humerus. Thereafter, the positioning vector of the origin of the ulna reference frame with respect to the bar frame, named $P_{\mathrm{Ulna}}$, was extracted from the homogeneous matrix $S{\mathrm{RF}}_{U}^{}$ and the position of the ulna origin with respect to the humerus reference frame was calculated:

PHUlna=SRFH−1·PUlna

The trajectory of the origin PHUlna identified the movement of the ulna with respect to the humerus (Figure 2C).

#### 2.3.2. Baseline Condition

**Figure 3:**
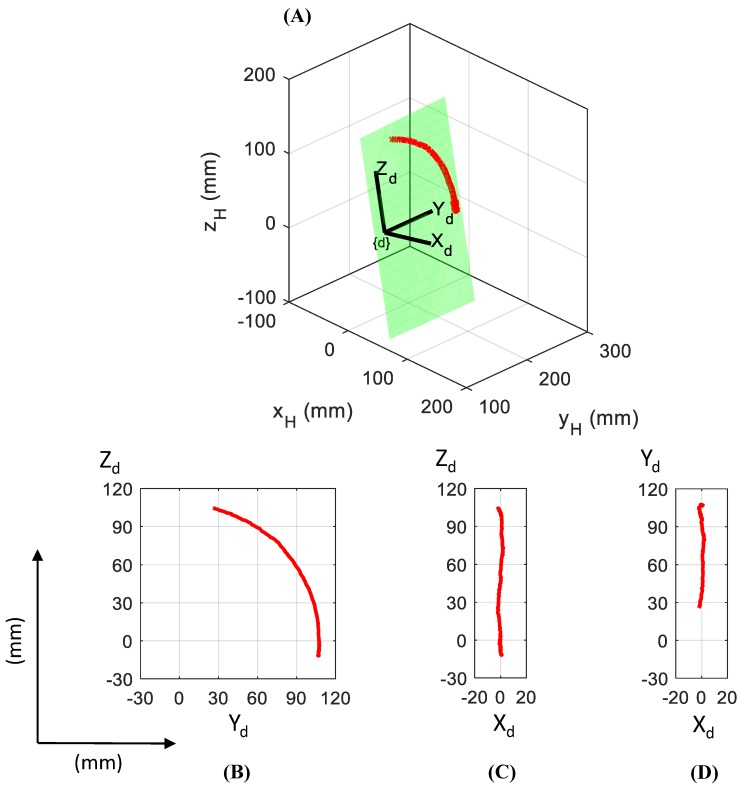
Baseline P_Ulna_ trajectory (red line) in 0–90° elbow flexion–extension referred to the humerus coordinate system (**A**) and in the deflection reference system planes Y_d_Z_d_, X_d_Z_d_, X_d_Y_d_ (**B**), (**C**), and (**D**). Green plane represents the regression plane.

The analysis of P_Ulna_ trajectory in an intact elbow during physiological flexion–extension motion (0–90°) defines a baseline condition for the results comparison. The red line in Figure 3 shows the displacement of the ulna origin during physiological flexion–extension motion. P_Ulna_ was expressed in the humerus coordinate system (x_H_y_H_z_H_ axes), and a regression plane of ulna motion was identified by means of a surface approximation. A deflection coordinate system (X_d_Y_d_Z_d_ axes) was assumed as fixed to the regression plane. The X_d_ axis oriented as orthogonal to the regression plane, Y_d_ was defined as the intersection between the regression plane and humerus plane x_H_y_H_, and Z_d_ axis was oriented orthogonal to the previous two axes. The origin of deflection coordinate system was positioned in the center of a circle obtained with the approximation of P_Ulna_ trajectory as arc of circumference. Figure 3 depicts a graphical representation of the regression plane (green plane) and P_Ulna_ path (red line), both in the humerus reference system (Figure 3A) and deflection coordinate system X_d_Y_d_Z_d_ (Figure 3B–D). The method used for the design of the deflection coordinate system infers the Y_d_ axis as representative of the flexion axis in one degree of freedom elbow model and the regression plane as representative of the elbow flexion plane.

#### 2.3.3. Maneuver Analysis

After identification of the regression plane in the baseline condition, trajectories of P_Ulna_ during orthopedic maneuvers were considered. The P_Ulna_ coordinates related to the humerus were identified from the ulna cluster of markers, then referred to the deflection coordinate system (X_d_Y_d_Z_d_) by means of transformation matrices. The elbow deflection was evaluated as the distance of P_Ulna_ to the elbow flexion plane. Finally, mean and standard deviation (STD) of the distance were calculated [24].

## 3. Results

**Figure 4:**
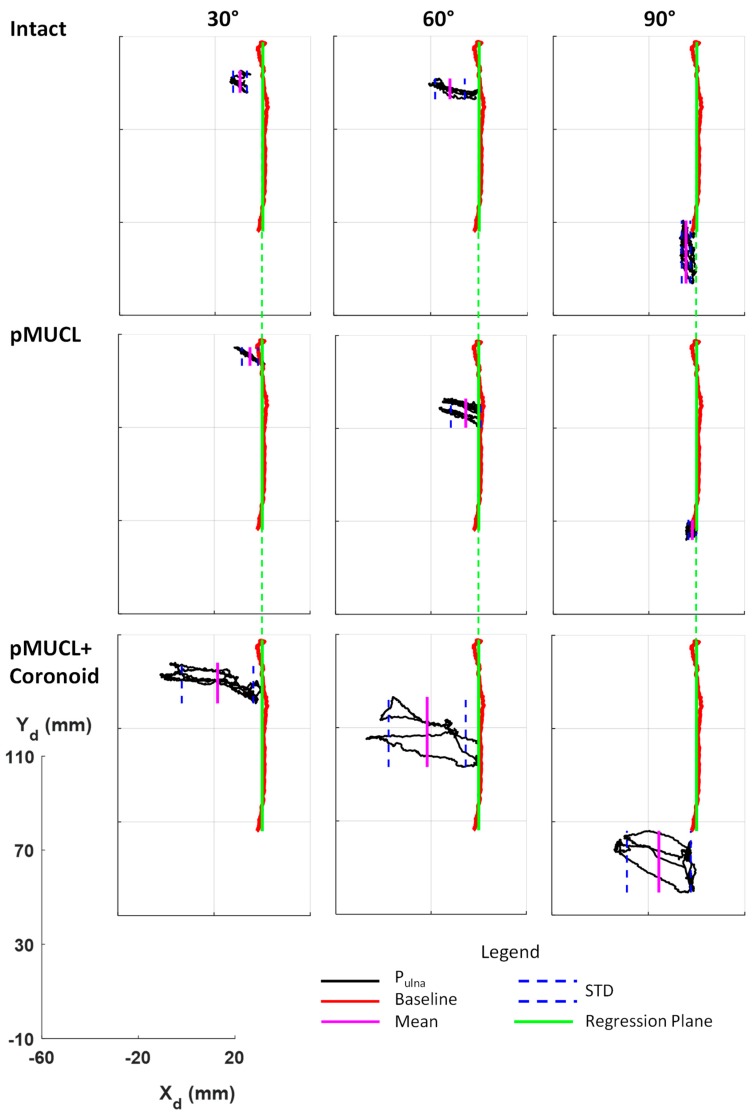
Elbow_1 stability for different surgical (intact, pMUCL section and pMUCL section + coronoid osteotomy) and biomechanical (static flexion of 30°, 60°, 90°) conditions are reported. The red line depicts the baseline trajectory of physiological elbow flexion–extension motion with an intact elbow and the green line represents the regression plane. The black line depicts the P_Ulna_ trajectory during the orthopedic maneuver with mean and STD values highlighted as pink and blue lines, respectively. A common reference axis is reported to describe the measures of deflection (mm).

**Figure 5:**
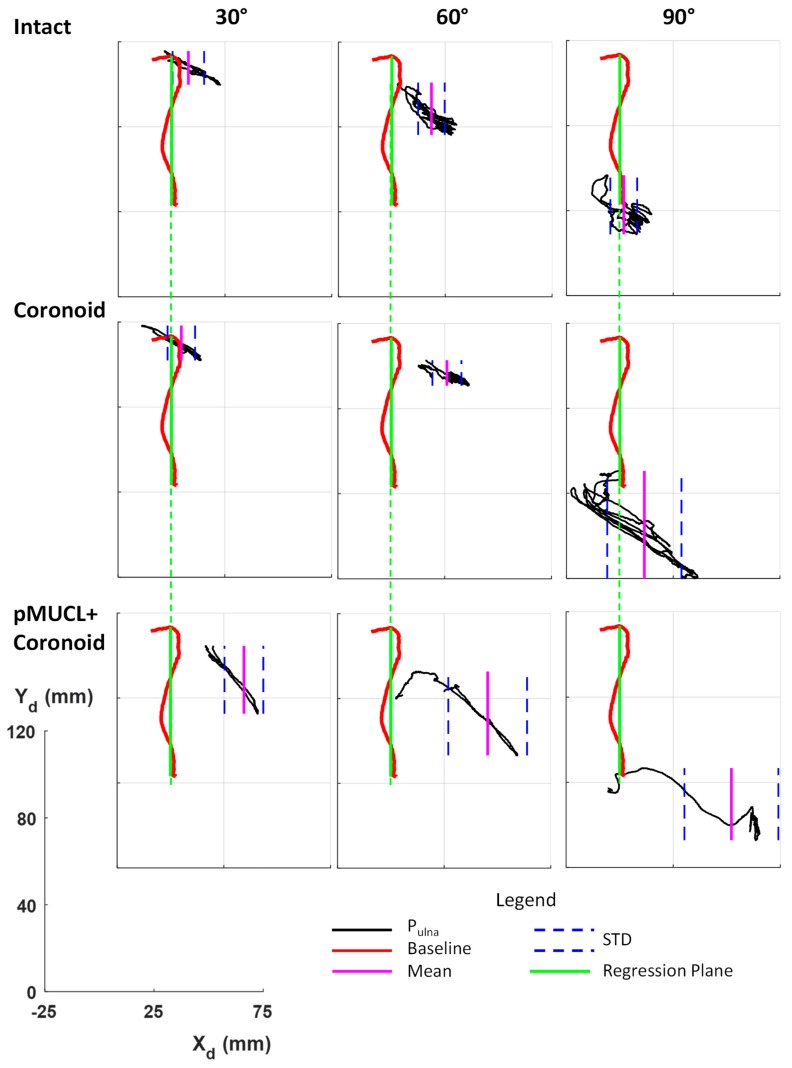
Elbow_2 stability for different surgical (intact, coronoid osteotomy and coronoid osteotomy + pMUCL section) and biomechanical (static flexion of 30°, 60°, 90°) conditions in transverse plane of the deflection coordinate system X_d_Y_d_. The red line depicts the baseline trajectory of physiological elbow flexion–extension motion with an intact elbow and the green line represents the regression plane. The lack line depicts the P_Ulna_ trajectory during the orthopedic maneuver with mean and STD values highlighted as pink and blue lines, respectively. A common reference axis is reported to describe the measures of deflection (mm).

Figure 4 and Figure 5 depict graphical representations of Elbow_1 and Elbow_2 results, respectively, while in Table 2 results are reported numerically. 

In Figure 4 and Figure 5, the trajectory of P_Ulna_ (black line) in all anatomical (intact elbow, pMUCL section and coronoid osteotomy) and biomechanical (static elbow flexion at 30°, 60°, 90°) conditions during orthopedic maneuver are reported in the transverse plane, considering the deflection coordinate system (X_d_Y_d_). The green line represents the regression plane calculated from the P_Ulna_ trajectory (red line) during the 0–90° flexion–extension elbow. Mean and standard deviation values of the distance of P_Ulna_ position to the regression plane are shown as a pink solid line and blue dashed line, respectively, in Figure 4 and Figure 5, while in Table 2 they are reported as numerical values.

## 4. Discussion

Based on reported graphical and numerical results found during the experimental tests, several considerations can be pointed out. Differences in deflexion direction and positive/negative mean values were obtained because the first specimen was a right upper limb, while the second was a left one. Moreover, due to differences in the anthropometric dimensions and consequently different marker bases positions, the direct comparison of values cannot be highlighted. As already underlined, in the adopted functional strategy, the loads application and the repetition reliability during maneuver was based on orthopedic experience, so force values were not measured. However, the overlapping of the two maneuver sequences in each case entitles us to assume the repeatability of the maneuvers. In Elbow_1, in the case of 60° flexion and the pMUCL section, the two sequences result at small different angles and can be easily distinguished, but it does not influence the ulna deflection with respect to regression plane. In Elbow_2, with both coronoid osteotomy and the pMUCL section, the maneuver was reported and considered in data elaboration only one time, because an error occurred during the data acquisition and not all 3D coordinates of all markers could be registered.

### 4.1. Intact Condition 

Comparing Elbow_1 and Elbow_2 at the intact condition, comparable trajectory trends characterized the two specimens. At 30° of elbow flexion, the MUCL ligament maintains the stability of the joint, while at 60° degree of flexion the elbow joint reveals a greater range of motion. The 90° flexion, due to the coronoid constraint, represents the angle with the most level of joint stability for both specimens. The highlighted results stress the different role of elbow joint constraints and the level of their intervention in elbow stability, both in the first and in the second specimen. The results reflect the expectations from the physiological and literature points of view. Considering the mean and STD values, the second specimen stresses a larger amount of ulna deflection compared to the first ones, which depict a higher level of joint stability. Probably, the different anatomy and strength level of constraints may cause these discrepancies.

### 4.2. First Section Condition (pMUCL) 

Considering the Elbow_1, the absence of the posterior bundle partly affects the elbow joint with a moderate instability at 30° and 60° of flexion, while at 90° the elbow results stable thanks to the coronoid process. Indeed, the ulna trajectory at 90° shows a similar trend compared to the intact ones (Figure 4). In Table 2, comparable mean (range from −1.81 to −12.04 mm) and STD (range 1.63–6.26 mm) values can be highlighted at all angles of flexion. In this case, the pMUCL is the only section that does not require the ligament reconstruction because the anterior bundle and the coronoid assure the elbow stability. Compared with previous studies that analyzed the absence of only the pMUCL, the present results do not confirm the gross instability of the elbow joint [18,21]. The analyzed variables in the current research referred to the ulna deflection distance from the approximated flexion plane (regression plane), instead of joint gapping and torsion angles, as investigated in previous studies [21]. This fact might be a possible reason which influenced the discrepancy in the results. Moreover, instead of the presence of the anterior bundle as a primary stabilizer, the posterior bundle’s role resulted secondary for the elbow stability.

### 4.3. First Section Condition (Coronoid) 

Concerning Elbow_2, in which the sequence of the section was inverted, the absence of coronoid influences the elbow stability at 90° (deflection = 11.49 ± 17.39 mm), with an increase of ulna deflection to regression plane during orthopedic maneuver. Small differences were registered at 30° (deflection = 4.64 ± 6.44 mm) and 60° (deflection = 26.05 ± 6.79 mm), compared to intact ones (deflection at 30° = 7.84 ± 7.4 mm, deflection at 60° = 18.52 ± 6.22 mm). In these cases, the ligaments maintain the elbow stability. Similar results were stressed by Shukla and colleagues, who confirmed the significant secondary stability of pMUCL in the setting of a coronoid fracture [23]. The increase of ulna deflection to the regression plane at all angles after pMUCL section reinforces the hypothesis of the significant importance and contribution of pMUCL to stability.

### 4.4. Coronoid + pMUCL Section Condition 

In both specimens, the contemporary section of pMUCL and coronoid osteotomy cause elbow instability. A large increase of deflection distance occurs at 30° (deflection Elbow_1 = −18.54 ± 14.89 mm, deflection Elbow_2 = 34.40 ± 9.10 mm), 60° (deflection Elbow_1 = −21.66 ± 16.2 mm, deflection Elbow_2 = 45.41 ± 18.47 mm) and 90° (deflection Elbow_1 = −15.63 ± 13.25 mm, deflection Elbow_2 = 52.16 ± 21.92 mm), as reported in Table 2. From the third column in both Figure 4 and Figure 5, it is possible to evaluate the large increase in distance of the ulna trajectory from the regression plane. Due to these results, the anterior bundle alone seems to be unable to maintain the stability. The reconstruction of the posterior bundle should be a good solution instead of a coronoid graft in case of an unfixable coronoid fracture. Gluck, Shukla and colleagues found similar results considering the same surgical condition but while evaluating distal and proximal gapping joints as variables of interest [19,23].

The present study points out the different roles of ligaments and bone constraints at several flexion angles. The coronoid process is a fundamental stabilizer for the elbow, especially at 90° of flexion. The posterior bundle role might be crucial in case of coronoid fracture, providing joint stability at lower flexion angles (30–60°). The absence of both coronoid and pMUCL caused the elbow gross instability, despite the intact anterior bundle. For all these reasons, surgeons could consider the reconstruction of the posterior bundle to limit the elbow instability. The biomechanical method of analysis allows overcoming anatomical differences between specimens. It might be a useful strategy to investigate elbow stability also in vivo, with a proper adaptation of the base plate to the human upper limb, thanks to the non-invasive characterization of clusters of markers. 

This preliminary study presents some limitations. The first limit can be identified in the small number and different size of specimens analyzed. A deeper investigation with a larger number of samples and different sequence of sections might be conducted in order to obtain significant and comparable results. With the attempt to directly compare results from different specimens, future work might be considered with the possibility to define the precise position of marker sets plates with respect to the elbow joint. As an alternative, values might be standardized with respect to the length of the upper limb. The replication of maneuver by means of several surgeons might be an analysis of interest in order to evaluate the inter-operator reliability, and it will be considered in future works. 

Moreover, the functional evaluation of the orthopedic maneuver instead of the external loads applied by means of a mechanical testing machine does not allow defining and measuring the force values, which can present differences between tests. This adopted solution might affect the repeatability of analysis between samples. Future works might be concentrated on the development of proper mechanical systems for measuring orthopedic applied forces and correct loads simulation among experimental tests.

As an alternative to the experimental test, the simulation process may be a promising solution. Through the multibody model of the human elbow, after a proper anatomical characterization based on a cadaveric sample, it is possible to investigate different combinations of ligaments and bone fractures, as to impose several biomechanical conditions. One possible future step might concentrate on the validation of the multibody elbow model already developed by means of comparison of experimental and computational results [15]. Moreover, the computational approach may be a useful solution to develop and analyze surgical reconstruction techniques. 

## Figures and Tables

**Table 1:** List of sequences of surgical section for Elbow_1 and Elbow_2.

Surgical Sections Sequence		
Test	Elbow_1	Elbow_2
Test 1	Intact	Intact
Test 2	pMUCL	Coronoid
Test 3	pMUCL + Coronoid	Coronoid + pMUCL

**Table 2:** Elbow_1 and Elbow_2 deflection results in terms of mean and standard deviation (STD) values.

P_ULNA_ Distance to Reference Regression Plane							
		Elbow_1			Elbow_2		
Elbow section		Intact	pMUCL	pMUCL + Coronoid	Intact	Coronoid	Coronoid + pMUCL
**Flexion angle**		30°					
**Elbow deflection**	Mean [mm]	−9.55	−5.09	−18.54	7.84	4.64	34.4
	STD [mm]	2.89	3.36	14.89	7.40	6.44	9.10
**Flexion angle**		60°					
**Elbow deflection**	Mean [mm]	−12.04	−5.42	−21.66	18.52	26.05	45.41
	STD [mm]	6.11	6.26	16.2	6.22	6.79	18.47
**Flexion angle**		90°					
**Elbow deflection**	Mean [mm]	−4.50	−1.81	−15.63	1.83	11.49	52.16
	STD [mm]	1.79	1.63	13.25	6.27	17.39	21.92
